# MFPSP: Identification of fungal species-specific phosphorylation site using offspring competition-based genetic algorithm

**DOI:** 10.1371/journal.pcbi.1012607

**Published:** 2024-11-18

**Authors:** Chao Wang, Quan Zou

**Affiliations:** 1 Center for Genomic and Personalized Medicine, Guangxi key Laboratory for Genomic and Personalized Medicine, Guangxi Collaborative Innovation Center for Genomic and Personalized Medicine, Guangxi Medical University, Nanning, Guangxi, China; 2 Institute of Fundamental and Frontier Sciences, University of Electronic Science and Technology of China, Chengdu, China; University of Auckland, NEW ZEALAND

## Abstract

Protein phosphorylation is essential in various signal transduction and cellular processes. To date, most tools are designed for model organisms, but only a handful of methods are suitable for predicting task in fungal species, and their performance still leaves much to be desired. In this study, a novel tool called MFPSP is developed for phosphorylation site prediction in multi-fungal species. The amino acids sequence features were derived from physicochemical and distributed information, and an offspring competition-based genetic algorithm was applied for choosing the most effective feature subset. The comparison results shown that MFPSP achieves a more advanced and balanced performance to several state-of-the-art available toolkits. Feature contribution and interaction exploration indicating the proposed model is efficient in uncovering concealed patterns within sequence. We anticipate MFPSP to serve as a valuable bioinformatics tool and benefiting practical experiments by pre-screening potential phosphorylation sites and enhancing our functional understanding of phosphorylation modifications in fungi. The source code and datasets are accessible at https://github.com/AI4HKB/MFPSP/.

## Author summary

Post-translational modifications (PTMs) is one of the key determinant factors of protein’s activity, stability, localization and folding. With the explosive growth of proteomics data, pre-screening of amino acid that with high potential to be phosphorylated is urgently needed before proceeding of wet experiment for the purpose of mechanism and function investigation. Although great progresses have been achieved in PTMs prediction, research on fungi has long been overlooked as algorithms suitable for fungal species PTMs identification are severely lacking. To fill this research gap, we developed a species-specific PTMs identification method, called MFPSP, for eight fungal species. In order to extract informative features, multiple sequence information was generated. The features were optimized by performing a global search strategy to avoid the local optima that traditional feature selection methods may encounter. Moreover, the effectiveness of MFPSP was proved by comparison with current excellent algorithms. It is expected our proposed model can effectively predict the PTMs of fungus and provide reliable candidates for further biological experiments.

## 1 Introduction

Phosphorylation, primarily occurs on serine (S), threonine (T) and tyrosine (Y) amino acid residues, is one of the most critical post-translational modifications (PTMs) that increase proteomic diversity through the regulation of protein activity, interactions and localization [1,2]. It has been reported that more than 30% of the mammalian proteins can be phosphorylated [3], and this ratio is updated to 75% in yeast and human proteomes [2,4]. The protein phosphorylation or dephosphorylation directly influences the signal transduction and various cellular behaviors, such as apoptosis, cell division, differentiation, and immune response [5]. Take fungus as an example, the biosynthesis of aflatoxins biosynthesis which causes food contamination word wide in *Aspergillus flavus* (*A*. *flavus*) is mediated by Fus3-MAP kinase phosphorylation module [6], titan cell formation and virulence in the fungal pathogen *Cryptococcus neoformans* (*C*. *neoformans*) is regulated by the phosphorylation of protein Gpa1 [7], FgSfl1 phosphorylation, in *Fusarium graminearum* (*F*. *graminearum*), is important for conidiation, sexual reproduction, and pathogenesis [8].

As the phosphorylation occurs on specific residual level, for a given protein, very few of the three residues can be phosphorylated, which implies efficient identification of the amino acid with high potential to be phosphorylated in a protein is essential, especially for further investigating its functional roles in cell biology and diseases as mentioned above. Although traditional wet experiment-based methods, such as radioactive labeling [9], LC-MS [10], and ChIP [11] had contributed greatly for this purpose, they are incompetent in the post-genomic era with exponentially growing data because of their time-consuming, laborious, and costly procedures [12]. Alternately, machine learning-based approaches[13,14], with advantages of high throughput, fast speed and low costing, are boosting for eliminating aforementioned experimental obstacles in PTMs identification [15].

Although innumerable tools have been developed for PTMs identification, most of them are designed for model organisms, especially for mammals, crops and industrial bacteria. Only a handful of reported methods are suitable for the prediction task of fungal species [16], and the performance achieved by existing in silico models still leaves much to be desired, which largely impedes the function investigation of PTMs in fungal proteins.

Here, we developed a novel tool, MFPSP, for phosphorylation site prediction in multi fungal species. Sequence characteristics were descripted by physicochemical features and distributed information, and an offspring competition-based genetic algorithm was applied for selecting the optimal feature subset. Finally, site specific model for seven fungal species were established independently. Independent testing demonstrated that our proposed model achieves a more advanced and balanced performance as compared to several state-of-the-art available toolkits.

**Fig 1:**
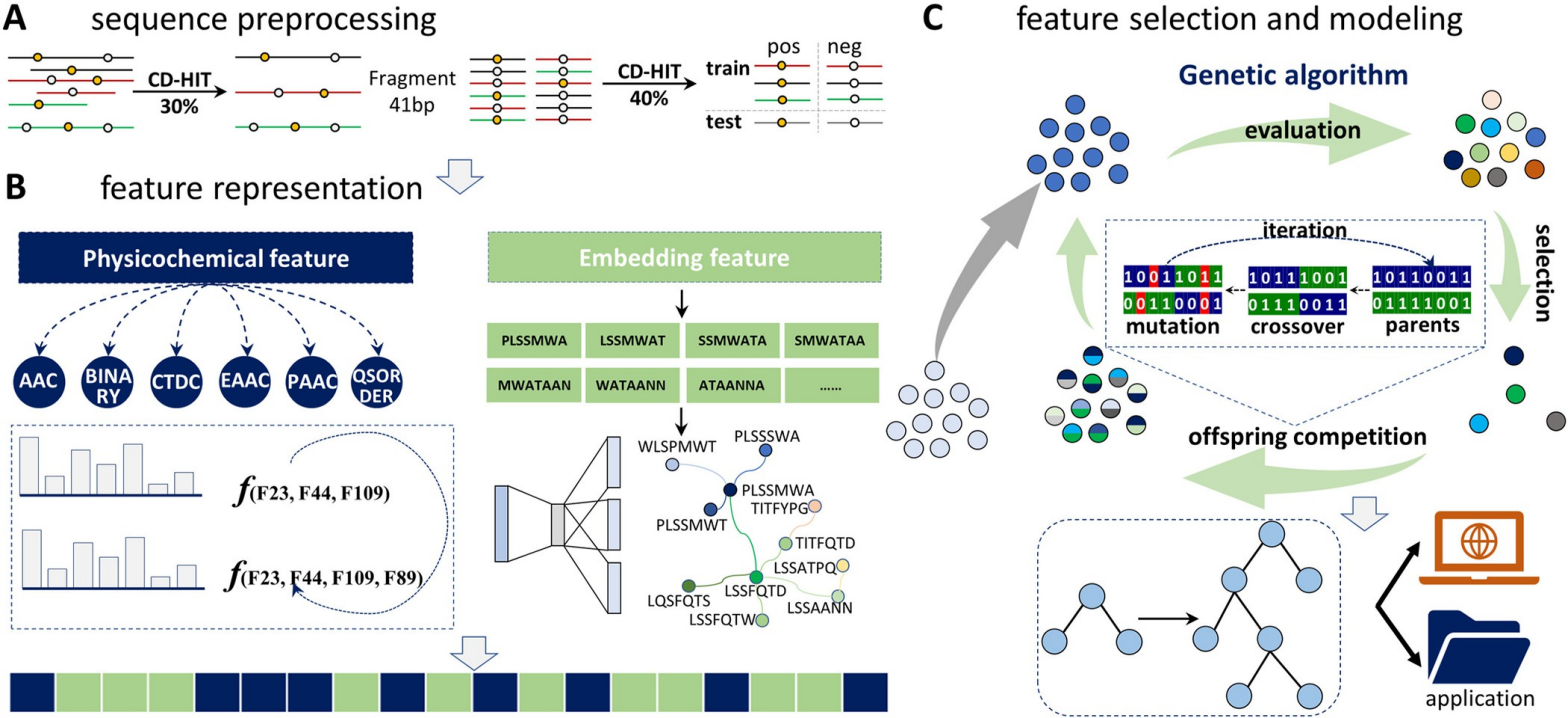
MFPSP workflow. A: sequence collection and redundancy reducing. B: feature representation by physicochemical and embedding methods. C: feature selection based on genetic algorithm with offspring competition and model construction.

Fig 1 illustrates the workflow of constructing the MFPSP model, which includes three main steps as described below.

## 2 Materials and methods

### 2.1 Data extraction and preprocessing

The fungi phosphorylation information of eight fungal species was retrieved from the Fungi Phosphorylation Database (FPD) [17]. Overall, 11222 proteins containing 62272 non-redundant phosphorylation sites were collected by FPD, the eight species are *A*. *flavus*, *Aspergillus nidulans* (*A*. *nidulans*), *C*. *neoformans*, *F*. *graminearum*, *Magnaporthe oryzae* (*M*. *oryzae*), *Neurospora crassa* (*N*. *crassa*), *Saccharomyces cerevisiae* (*S*. *cerevisiae*) and *Schizosaccharomyces pombe* (*S*. *pombe*). The number of proteins and phosphorylation S/T/Y sites for each species are listed in Table 1.

**Table 1:** The statistics of number of proteins and phosphorylation S/T/Y sites for different organisms.

Species	Site type	Proteins	Proteins (sim 0.3) ^#^	Fragments (pos/neg)	Fragments (sim 0.4) (pos/neg)
*Aspergillus sp*.^*^	S	794	619	1086/44199	673/10647
	T	301	239	310/11587	235/3922
	Y	41	36	36/638	—
*C*. *neoformans*	S	613	601	1214/42669	636/9586
	T	186	179	249/7529	166/2649
	Y	20	19	20/256	—
*F*. *graminearum*	S	1343	1322	2874/83705	1838/20178
	T	576	569	766/25889	603/8702
	Y	67	67	71/1300	—
*M*. *oryzae*	S	1415	1383	3759/86208	1872/20943
	T	601	405	680/17844	447/6261
	Y	23	21	23/49	—
*N*. *crassa*	S	1529	1516	3614/106281	2369/24519
	T	812	805	1288/39062	992/12656
	Y	137	136	151/2852	—
*S*. *cerevisiae*	S	3880	3323	25495/151792	9535/38748
	T	2789	2440	8563/79890	4937/27925
	Y	1284	1193	1815/24888	1365/11899
*S*. *pombe*	S	1316	1225	3049/73498	1781/16254
	T	409	386	567/13718	434/4916
	Y	82	75	81/1595	—

^*^ *A*. *flavus+A*. *nidulans*

^*#*^ sim: sequence similarity

A strict redundancy reducing procedure was applied for proteins and peptide fragments, which is detailed in [18]. In brief, protein redundancy was eliminated to a threshold of 0.3, then 41 bp length of peptide fragments were extracted with S/T/Y in the center [19,20]. At last, the redundancy of extracted fragments was further reduced to a threshold of 0.4. To construct a balanced dataset, the negative samples were randomly sampled to same number of the positive. Finally, 80% samples in above the balanced dataset were used for training, the remaining 20% were used for testing.

### 2.2 Feature representation

Representing the protein fragment into discriminative feature is crucial for reliable and superior model. In this work, two types of feature-encoding algorithm were applied to enrich the fragment information.

### Sequence physicochemical-based features

Five feature descriptors aiming to formulate the physicochemical property of protein fragment were employed for all species. These features are amino acid composition (AAC), C/T/D-composition (CTDC), enhanced amino acid composition (EAAC), pseudo-amino acid composition (PAAC), quasi-sequence-order (QSOrder). In addition, the BINARY method was selectively used since which showed better performance in larger dataset. They are described in detail in the S1 Methods.

### Embedding-based features

Recently, embedding algorithms, e.g., FastText, Glove and Word2vec, have been widely used for distributed representation of all kinds of biological sequence for the downstream task of classification [21–24], clustering [25,26], gene-disease or protein-protein interaction [27,28], and so on [29]. In the framework of FastText, embedded feature for each word in a vocabulary is shaped by its context information, where similar words are close in spatial distribution, and each word was represented as a predefined n-dimensional numeric vector [30]. The process for protein fragment with N amino acid residues embedding is briefly described as follows. The protein fragment was first transferred into a bio-sentence in an overlapping manner by sliding k (k<N) length window along the sequence with a stride length of 1, each generated k-mer was regard as a word in this bio-sentence. Then, the FastText was applied to embedding each word into a fixed 20-dimensional numeric vector by adopting the skip-gram model in this work. Each fragment feature was represented by sequentially concatenating feature of each word in the sequence, which is a vector of size (N-k+1)×20. The Gensim library (v4.2, https://radimrehurek.com/gensim/) was employed to implemented the FastText framework.

### 2.3 Feature selection based on genetic algorithm with offspring competition

Constructing machine learning model directly on the above physicochemical-based features and embedded features may result sub-optimal performance in view of the information redundancy. Hence, in this study, genetic algorithm [31] was applied to screen out the optimal feature subset form the combined features. The major framework of genetic algorithm was reported in our early works [32], the offspring selection process was improved by a competition strategy to advance algorithm’s efficiency. The process is briefly described as follows. First, a constant number of populations (feature subsets) were randomly generated from the original features and each of the subset, i.e., the chromosome in genetic algorithm, is constrained to 100D. Then, the feature subsets were evaluated by a specified fitness function, subsets that showed a better performance were selected as the parents to generate new populations (offspring) by three genetic operators, selection, crossover, and mutation.

The selection process was optimized by offspring competition strategy. The fitness value was sorted by a descending order and the first third of top feature subsets were choose for offspring competition. For each pair of parents, a two-point crossover method was used to generate offspring, followed by a random mutation with probability of 0.0003. To avoid prematurity, the crossover procedure operated 10 times and the number of mutation times increased once every five generations. As only a third of the populations were selected for the offspring competition, the selection step implemented three times to generate a same population. Two global parameters, the number of population and generation was set to 60 and 150, respectively.

### 2.4 Model training and evaluation

Five metrics were used to comprehensively measure the performance of the ensemble model: ACC, specificity (SP), sensitivity (SN), Matthews correlation coefficient (MCC), and AUC. They were calculated as follows:

$$ACC=\frac{TP+TN}{TP+TN+FP+FN}$$ (1)

$$SN=\frac{TP}{TP+FN}$$ (2)

$$SP=\frac{TN}{TN+FP}$$ (3)

$$MCC=\frac{TP\times TN-FP\times FN}{\sqrt{\left(FP+TP)(FN+TP)(FP+TN)(FN+TN\right)}}$$ (4)

The metric AUC calculates the area under the receiver operating characteristic curve based on the false-positive rate (FPR) and the true positive rate (TPR) under various thresholds. The TPR and the FPR were calculated as follows:

$$TPR=\frac{TP}{TP+FN}$$ (5)

$$FPR=\frac{FP}{TN+FP}$$ (6)

where TP = true positive, FP = false-positive, TN = true negative, and FN = false negative. SN and SP were employed to evaluate the model performance with respect to the positive and negative samples, respectively. The remaining three metrics are global prediction performance indicators.

## 3 Results and discussion

### 3.1 Descriptor parameter optimization and feature selection

Eight fungal species are included in this study, and the feature optimization and model building process are similar. In order to make the description concise and explicit, the serine phosphorylation of *S*. *cerevisiae* was taken as an example (except where noted) in the following context. Five physicochemical descriptors were applied for sequence physicochemical property representation, and three of them, i.e., Qsorder, EAAC, and PAAC, were conducted for parameter optimization to make each of them as informative as possible. The parameter search range, evaluation metrics and the optimal value are listed in S1 Methods and S1 Table.

Based on the optimal parameters determined above, sequences were numerically formulated by the five feature algorithms. Considering the high feature dimension generated by of EAAC and BINARY descriptor, the top 250 most importance features calculated by light gradient boosting machine (LGB) method of the two methods were roughly selected for feature combination. Finally, a feature subset with dimensionality of 623 were generated. Of note, 373D features were generated for several species where the BINARY descriptor was not used as mentioned in materials and methods.

**Fig 2:**
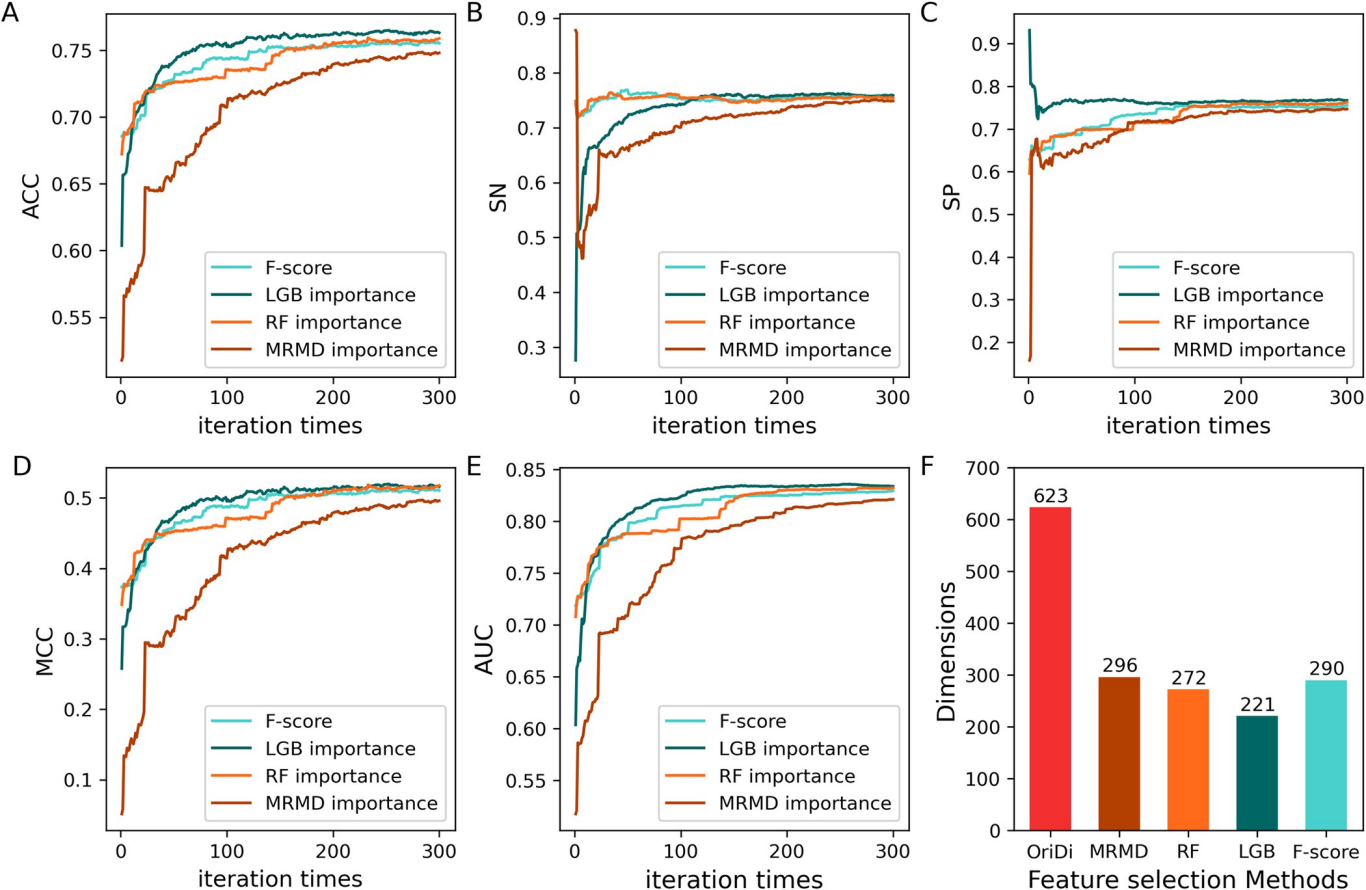
Comparison results of four feature selection strategies for *S*. *cerevisiae* S phosphorylation site. OriDi: original feature dimension.

For serine phosphorylation of *S*. *cerevisiae*, the 623D features were further optimized by sequential forward search (SFS) method as elaborated in [33]. Four different feature importance list calculated by F-score, LGB, MRMD [34] and random forest (RF), respectively, were subjected to SFS feature selection based on SVM algorithm (S1 Methods and S2 Table). The comparative results were showed in Fig 2. Generally, values of the five metrics increase rapidly in the top 100 important features, and then tend to be flat with more features added to the model. In terms of the four types of feature importance ranking methods, the LGB-based method resulted the best performance when evaluated by metric ACC, SP, MCC and AUC, the MRMD-based methods exhibited lowest efficiency (Fig 2A–2E). The optimal feature subset was obtained based on the AUC value, which resulted in a feature subset of 221D with AUC of 0.8342. As depicted in Fig 1F, the LGB-based method filtered out approximately 65% of the original 623D features, which also the most effective among the four methods. Collectively, our results indicated that the original combined features were serve redundancy, the LGB-based SFS feature optimization strategy is superior than others and adopted for the final optimal feature selection.

### 3.2 Distributed representation of nucleic acids fragments

**Fig 3:**
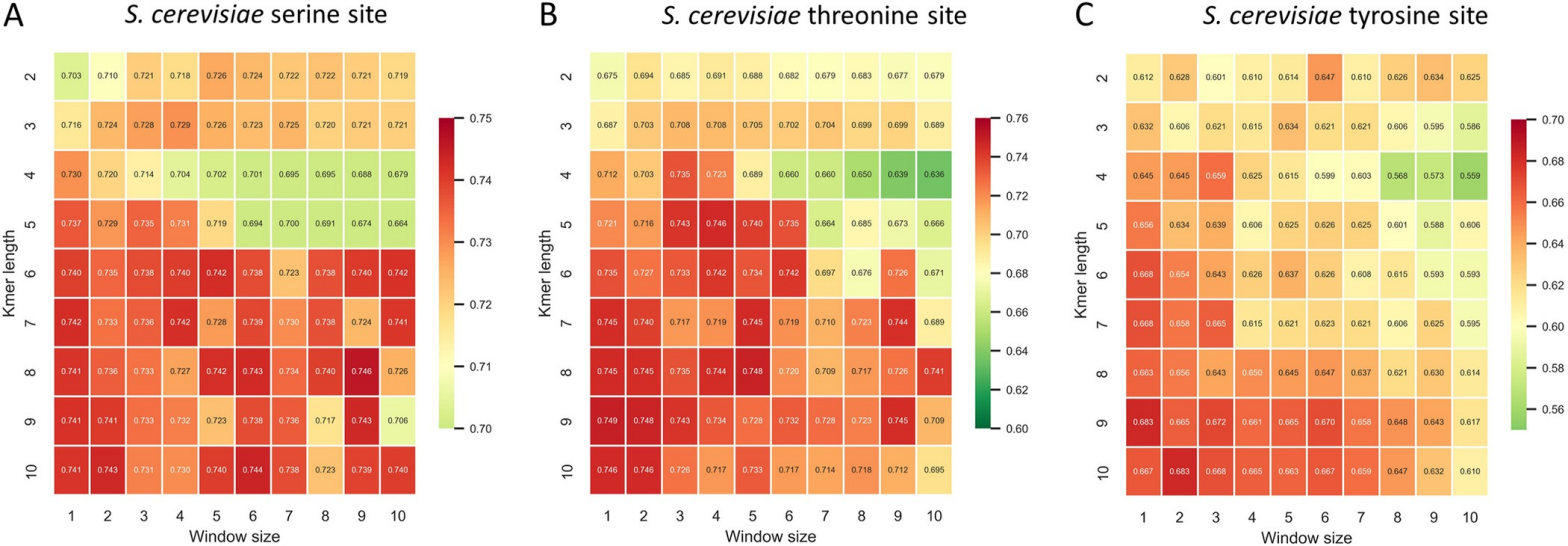
Accuracy values of the model constructed with embedded features with different k-mer length and window size.

To further enrich the sequence information, the word embedding algorithms were employed to mining the semantic information. Each k-mer in the corpus was embedded into vector with 20D, and the sequence feature vector was generated by sequentially concatenating feature of each word in the sequence. Two critical parameters of FastText, the k-mer length K (from 2 to 10) and window size W (from 1 to 10), were optimized by a grid search method (S1 Methods and S4 Table). As depicted in Fig 3, the value of K exhibited a more decisive impact on the ACC. Models that with small K and W are more in favour when their ACC values showed no significant difference. Taken together, k-mer length of 7 and a window size of 1 were adopted for the final embedding model of *S*. *cerevisiae* S site (Fig 3A). Based on the optimal K and W, 700D features were generated, then a same SFS procedure was applied to this embedding vector and, finally, 278D were remained. For *S*. *cerevisiae* T (K = 7 and W = 1) and Y site (K = 9 and W = 1) (Fig 3B–3C), there were 292D and 172D features retained, respectively after SFS optimization.

### 3.3 Feature redundancy eliminating based on genetic algorithm with offspring competition strategy

As stated above, for *S*. *cerevisiae* S site, the physicochemical feature (623D) and the embedded feature (700D) were reduced to 221D and 278D, respectively using the SFS method based on LGB feature importance. During this feature optimization process, feature was added one by one to training prediction model. However, this approach somewhat neglected the comprehensive consideration of feature combinations and global-level optimization, Hence, we embarked on eliminating redundant features more rigorously.

In the present study, the genetic algorithm was employed to screen out the optimal feature combination from the combined 499D features (S1 Methods), the framework of the genetic algorithm is detailed in [32]. To avoid premature convergence phenomena, the algorithm’s selection operator was optimized by an offspring competition strategy (see material and methods). Of note, the generation of genetic algorithm was set to 150, and, for comparative purpose, the top 2 most effective feature selection strategies i.e., LGB and F-score based methods, exhibited in Fig 2, were chosen to reduce the feature dimensionality.

**Fig 4:**
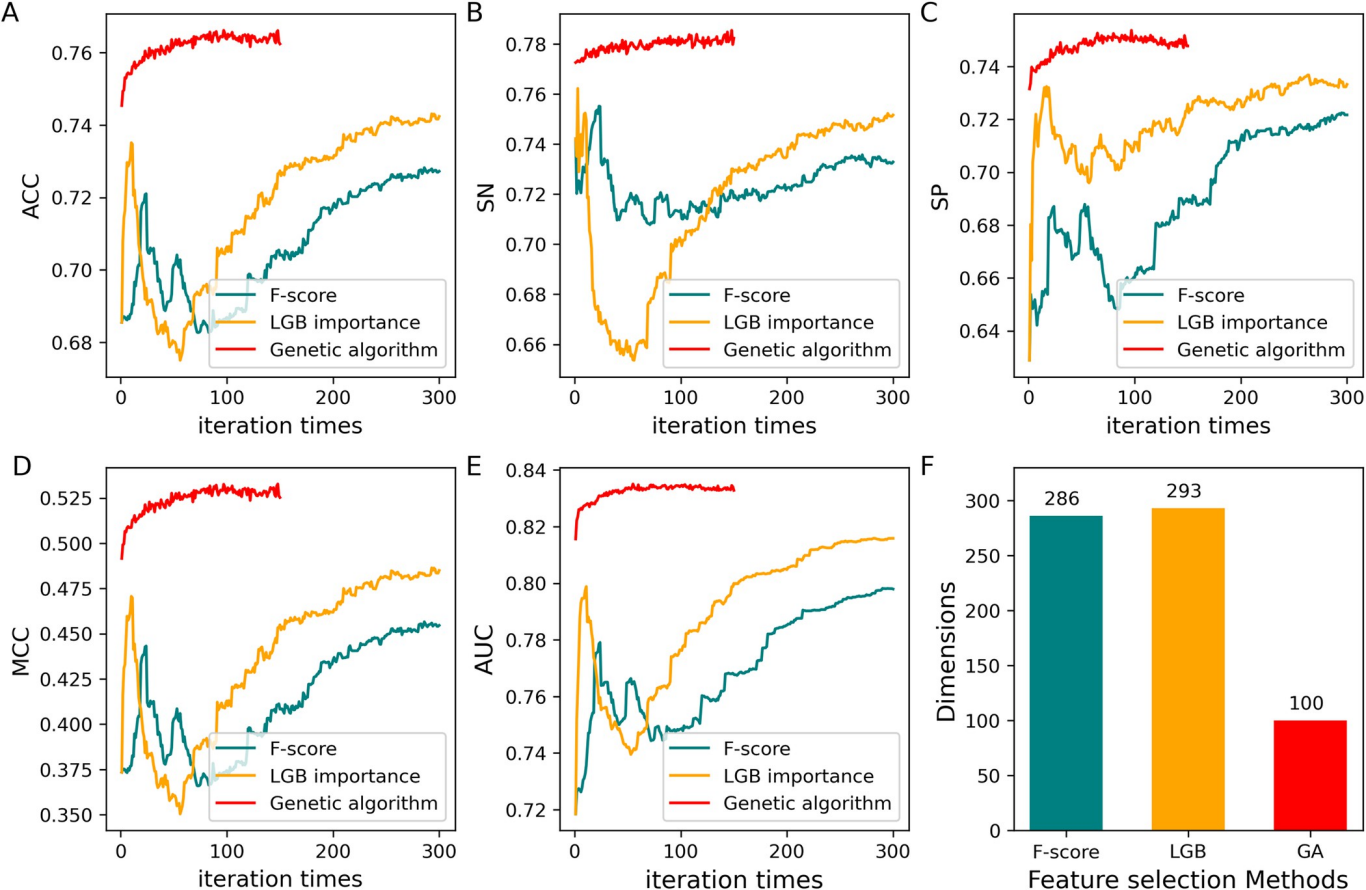
Performance comparison of feature selection strategies for *S*. *cerevisiae* S phosphorylation site.

As shown in Fig 4 for *S*. *cerevisiae* S site, the feature dimension of the genetic algorithm is fixed to 100 as the demand of algorithm framework, while that of LGB and F-score based method is linearly increased with iteration times. Apparently, the genetic algorithm significantly outperformed the comparison methods in all evaluation metrics (Fig 4A–4E). Overall, in light of metrics and dimension (Fig 4F), it can be stated that the offspring competition-based genetic algorithm is superior than others in feature optimization. The detailed metrics for each of the final optimal model are listed in Table 2.

**Table 2:** The prediction performance for the fungi phosphorylation S/T/Y site in seven organisms.

Residual type	Fungal species	ACC	SN	SP	MCC	AUC
S	*Aspergillus sp*.	0.8159	0.8131	0.8187	0.6324	0.8725
	*C*. *neoformans*	0.8402	0.8373	0.8431	0.6816	0.9014
	*F*. *graminearum*	0.8298	0.8329	0.8268	0.6602	0.8910
	*M*. *oryzae*	0.8495	0.8518	0.8471	0.6990	0.9060
	*N*. *crassa*	0.8681	0.8839	0.8522	0.7368	0.9365
	*S*. *cerevisiae*	0.7613	0.7775	0.7452	0.5231	0.8335
	*S*. *pombe*	0.8214	0.8056	0.8372	0.6435	0.8955
T	*F*. *graminearum*	0.8033	0.7888	0.8179	0.6083	0.8668
	*M*. *oryzae*	0.8179	0.8317	0.8040	0.6366	0.8701
	*N*. *crassa*	0.8398	0.8663	0.8133	0.6811	0.9156
	*S*. *cerevisiae*	0.7532	0.7608	0.7456	0.5066	0.8199
	*S*. *pombe*	0.8175	0.8017	0.8330	0.6377	0.8797
Y	*S*. *cerevisiae*	0.7202	0.7005	0.7399	0.4412	0.7789

### 3.4 Feature intersection, contribution and pattern analysis

To look deeper into features that shared among different species, we processed the comparison in five models that adopted the same K and W parameters for distributed representation of S phosphorylation site. As depicted in S1 Fig, nearly 40% of the total 100 features are shared by at least one other model. For example, 38 features of *S*. *cerevisiae* were presented in other 4 species, where ten of them were identical with *S*. *prombe* and *F*. *graminearum*, 6 of them were identical with *M*. *oryzae*.

**Fig 5:**
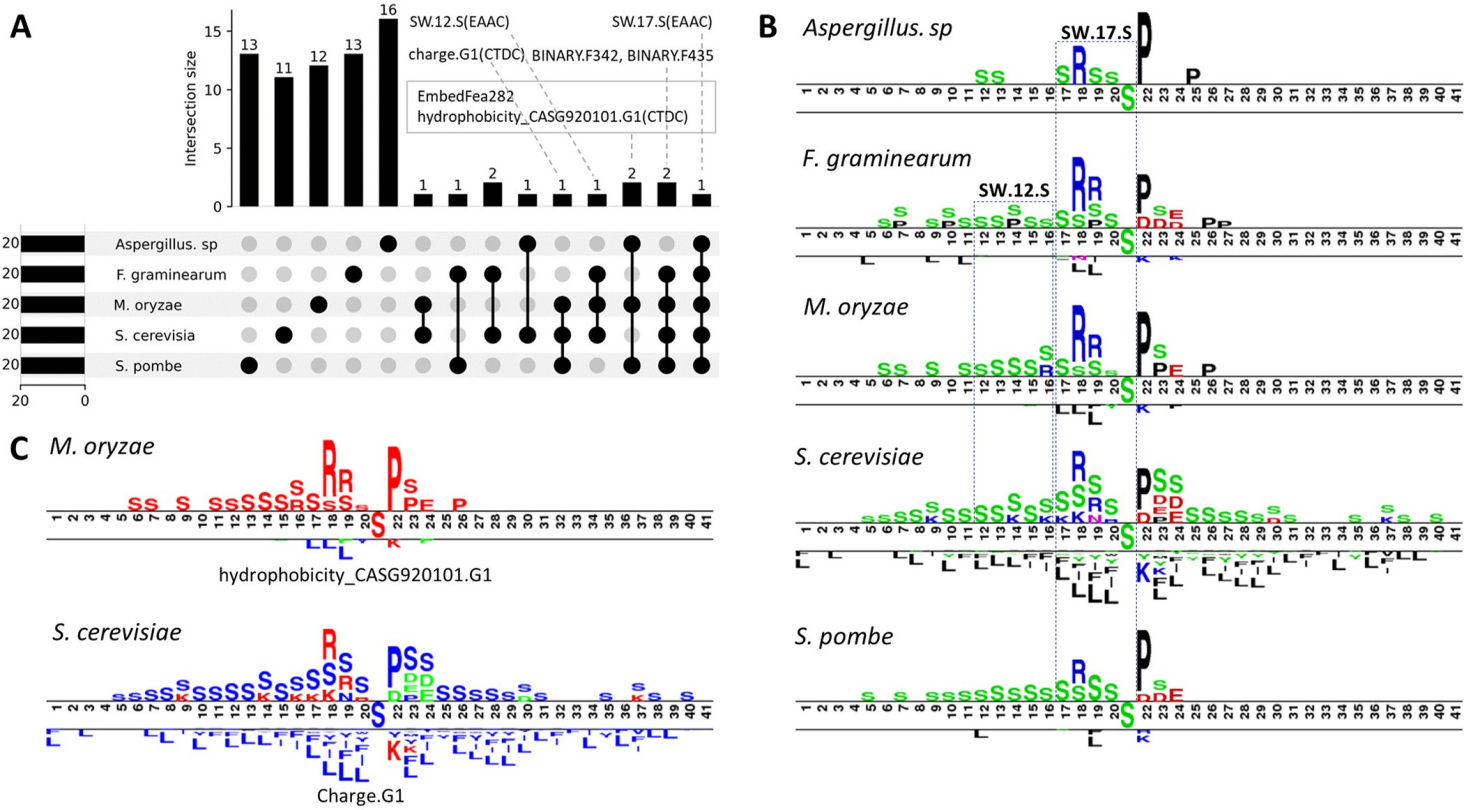
Feature intersection and sequence patterns for *S*. *cerevisiae* S site of five fungal species. The enrichment and depletion bias of amino acid was calculated by Two-Sample-Logos (http://www.twosamplelogo.org/).

We then specially focused on the top 20 most important features inferred by SHAP [35–37]. The feature intersection and sequence patterns were exhibited in Fig 5. There are seven features shared by at least two species, SW.17.S (EAAC) is the only one that identified in all species. Based on the calculation formula [38], for a 41bp amino acid fragment, SW.17.S(EAAC) refers to the S composition in 17th sliding 5-mer window, namely 17 to 21 bp in the fragments, which was in line with the amino acid enrich and deplete biases pattern as shown in Fig 5B, similar pattern was also observed for feature SW.12.S(EAAC) which presented in three species. BINARY.F342 refers to composition of arginine (R) at the 18th position in the 41bp fragments, significant amino acid biases can be seen at this position in all five species (Fig 5B). Another obvious amino acid enrich pattern, corresponding to BINARY.F435, was the Proline (P) at 22th position in the fragments. The latter two features generated by BINARY descriptor were kept in four of the five species, suggesting the discriminative ability of features relation to sequence composition patterns. Charge.G1(CTDC) and hydrophobicity_CASG920101.G1(CTDC) refers to charge (K and R) and polar (“KDEQPSRNTG”) properties, respectively (Fig 5C). EmbedFea282 corresponds to the embedded feature of the fifteenth 7-length k-mer, namely 15 to 21bp in the fragments, which partially explains the enrichment of various feature patterns in this region as mentioned above. Overall, these results indicated that each type feature contributed uniquely to the augmentation of sequence information, and our feature optimization strategy is competent to mining and retain the informative characteristic in sequence.

**Fig 6:**
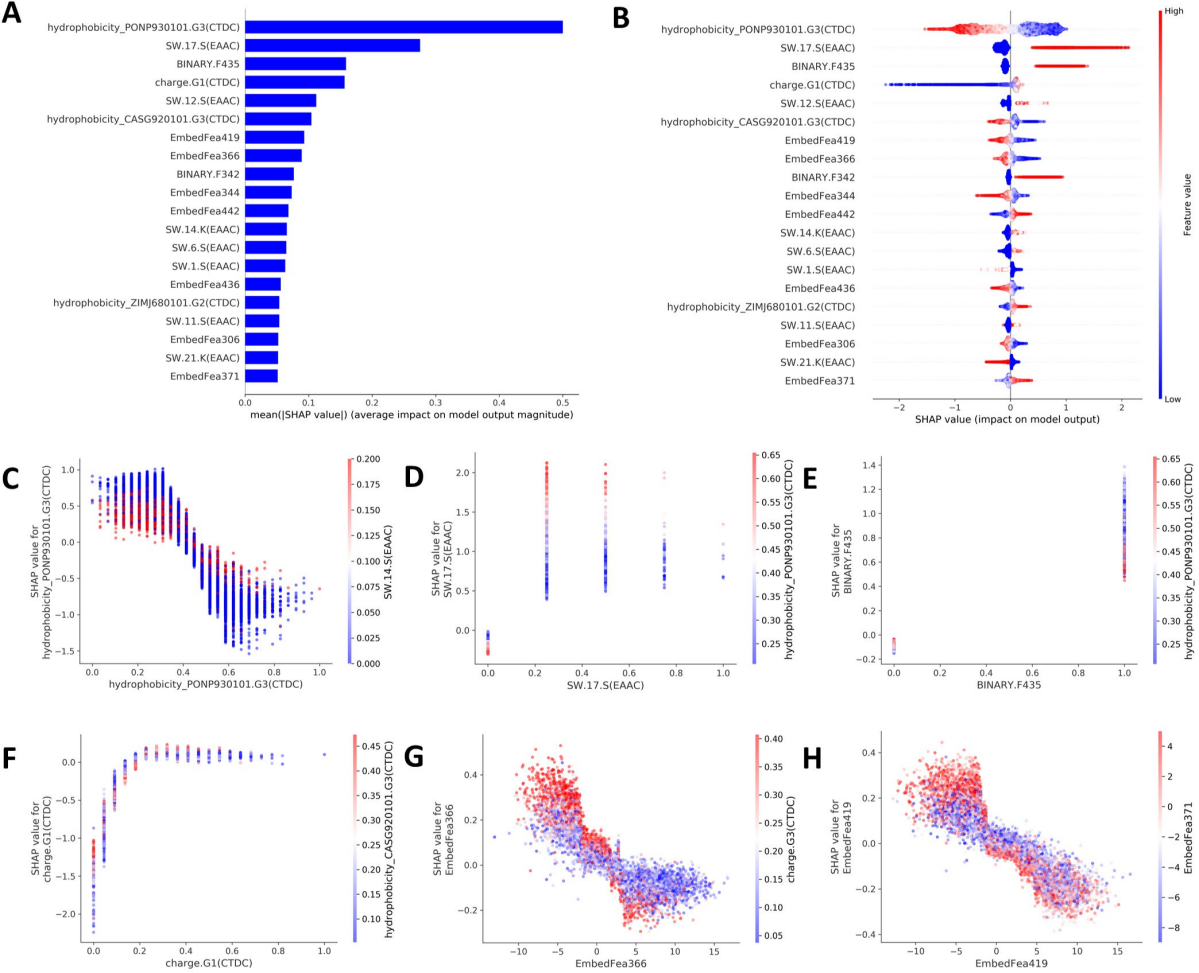
Feature importance, contribution and dependency analysis. A: the 20 most important features. B: summary plot for feature value contribution. The x-axis represents the SHAP values, representing the impact that feature had on the model’s performance. C–H: SHAP dependence plots. These plots show the effect that a single feature has on the model and the interaction effects across features.

A further exploration of contribution of features have on the mode’s performance was processed based on SHAP. For feature importance, hydrophobicity_PONP930101.G3(CTDC) contributed most to the S phosphorylation, followed by SW.17.S(EAAC) and BINARY.F435. Seven of the top 20 most important features were generated by embedding method, suggesting the excellent efficiency of this feature representation strategy (Fig 6A). The decisive trend for each feature value were showed in Fig 6B, where higher value for 11 features presented a positive impact on model behaver, the remaining nine features showed contrary impacts. For example, a lower value of hydrophobicity_PONP930101.G3(CTDC) is associated with positive impact on S phosphorylation identification, while lower of this value exhibited opposite effect. It is worthy of note that the top share features and patterns, such as SW.17.S, SW.12.S (Fig 5), are also the contributed most for model performance (Fig 6B).

Moreover, we tried to further explore the possible mechanisms of how these features correlate with biological functions. Our results showed that the R and S site (SW17.S) was significantly enriched near left of S phosphorylation site, indicating that the intrinsic disorder of protein, promoted by R, K, E, P and S sites, are crucial for the phosphorylation [39]. It was reported that phosphorylation on hydrophobic motif creates a specific docking site that recruits and activates phosphoinositide-dependent kinase-1, which then activates the downstream actions [40]. Residual charge, such as Charge.G1, is also an important factor for phosphorylation, all-atom molecular dynamics simulations demonstrated that distribution positively charged residues throughout the protein sequence has great impact on salt bridge formation, which influences the effect of phosphorylation [41].

Finally, deeper insight into interaction effect, calculated by SHAP dependence plot, of the top 20 features were presented in Figs 6C–6H and S2. Feature turning point, complement to Fig 6B, can be visualized. For example, the turning value for feature hydrophobicity_PONP930101.G3(CTDC) is approximately 0.4, feature value higher than that threshold weakened model behavior, for while value lower than that resulted in contrary effect (Fig 6C). Charge.G1(CTDC) exhibited an opposite trend, where the turn point is approximately 0.2, and values higher than that change SHAP values from negative to positive (Fig 6F). Excepting their independent contribution, interplay between features are widely exist. Interaction between low EmbedFear366 values (range-10 to 2.5) and high Charge.G3 (CTDC) values (range 0.2 to 0.4) exhibited a prompt impact on model behavior (SHAP values>0), but the latter showed little impact when EmbedFear366 values beyond 2.5 (Fig 6G). A high feature value of SW.17.S (0.0 to 1.0) were positively correlated with the value of hydrophobicity_PONP930101.G3(CTDC) (Fig 6D), while the later showed a negative interaction with BINARY.435 (Fig 6E). More feature interaction patterns can be seen in Fig 6C–6H and S2.

### 3.5 Comparison with existing predictors

Considering the representativeness and availability, four methods were selected for the comparison, including the general model NetPhos (v 3.1) [42], yeast-serine and threonine specific model NetPhosYeast [43], plant-specific tool PHOSER [44] and ScerePhoSite [18] that designed for *S*. *cerevisiae* (see more detail in S5 Table).

Model performance evaluation and comparison were processed on the independent testing sets. The detailed comparative results were presented in Table 3. Our proposed model MFPSP, on the whole, achieved the best metrics on ACC, SN, MCC and AUC in nearly all fungi species and phosphorylation sites. For example, MFPSP resulted in highest ACC (0.7754), SP (0.7536), MCC (0.5512), AUC (0.8580) on *Aspergillus sp*. S site, with an increase of ACC in range of 4 to 16%, SP in range of 4.7 to 72.3%, MCC in range of 7 to 13.2%, AUC in range of 1.5 to 13.2%, respectively, compared with the other three methods. Of note, another strength of the MFPSP is the unbiased prediction on positive and negative phosphorylation sites, the value |SN-SP| varies in range of 0.73 to 6.56, while the compared methods are seriously biased. For instance, although NetPhosYeast achieved the best SP (0.9347) on *Aspergillus sp*. S site, the SN of this method is 0.0297, which resulted in a |SN-SP| value of 90.50%. This suggests that NetPhosYeast predicts almost all query sequences as non-phosphorylation site. Opposite prediction bias can also be observed, such as NetPhos for *S*. *cerevisiae* S site with a |SN-SP| value of 52.60%, where the prediction result is seriously skewed to be phosphorylable. Collectively, these results demonstrate that MFPSP is significantly superior than the existing methods for fungi phosphorylation site identification.

**Table 3:** Performance comparison of MFPSP with existing predictors on independent test data.

Residual type	Fungal species	Method	ACC	SN	SP	|SN-SP|%	MCC	AUC
S	*Aspergillus sp*.	NetPhos	0.5978	0.2826	0.9130	63.04	0.2520	0.7257
		NetPhosYeast	0.6159	0.0297	**0.9347**	90.50	0.3010	0.7931
		PHOSER	0.7355	0.6304	0.8405	21.01	0.4817	0.8431
		MFPSP	**0.7754**	**0.7536**	0.7971	**4.35**	**0.5512**	**0.8580**
	*C*. *neoformans*	NetPhos	0.6190	0.2777	0.9603	68.26	0.3257	0.7297
		NetPhosYeast	0.6865	0.4047	**0.9682**	56.35	0.4515	0.8433
		PHOSER	0.7777	0.6349	0.9206	28.57	0.5797	**0.8938**
		MFPSP	**0.8333**	**0.8651**	0.8015	**6.36**	**0.6680**	0.8896
	*F*. *graminearum*	NetPhos	0.5765	0.2240	0.9289	70.49	0.2157	0.7340
		NetPhosYeast	0.6256	0.3114	**0.9398**	62.84	0.3231	0.7877
		PHOSER	0.7254	0.6229	0.8278	20.49	0.4605	0.8005
		MFPSP	**0.8278**	**0.8607**	0.7951	**6.56**	**0.6572**	**0.8859**
	*M*. *oryzae*	NetPhos	0.5949	0.2887	0.9010	61.23	0.2401	0.6948
		NetPhosYeast	0.6524	0.3716	**0.9331**	56.15	0.3683	0.8021
		PHOSER	0.7713	0.7085	0.8342	12.57	0.5471	0.8447
		MFPSP	**0.8316**	**0.8556**	0.8074	**4.82**	**0.6639**	**0.9047**
	*N*. *crassa*	NetPhos	0.5917	0.2510	0.9324	68.14	0.2507	0.7163
		NetPhosYeast	0.6582	0.3691	**0.9472**	57.81	0.3878	0.8221
		PHOSER	0.7215	0.6455	0.7974	15.19	0.4482	0.7947
		MFPSP	**0.8618**	**0.8755**	0.8481	**2.74**	**0.7239**	**0.9343**
	*S*. *cerevisiae*	NetPhos	0.5723	**0.8353**	0.3093	52.60	0.1701	0.6425
		NetPhosYeast	0.6397	0.8154	0.4640	35.14	0.2985	0.7255
		PHOSER	0.6961	0.6303	0.7619	13.16	0.3956	0.7600
		ScerePhosSite	0.7595	0.7488	**0.7703**	2.15	0.5193	**0.8406**
		MFPSP	**0.7614**	0.7714	0.7514	**2.00**	**0.5229**	0.8397
	*S*. *pombe*	NetPhos	0.6067	0.3117	0.9016	58.99	0.2643	0.7068
		NetPhosYeast	0.6769	0.4129	**0.9410**	52.81	0.4167	0.8124
		PHOSER	0.7359	0.6882	0.7837	9.55	0.4740	0.8137
		MFPSP	**0.8062**	**0.7893**	0.8230	**3.37**	**0.6127**	**0.8801**
T	*F*. *graminearum*	NetPhos	0.6041	0.4166	0.7916	37.5	0.2247	0.6401
		NetPhosYeast	0.6583	0.6666	0.6500	**1.66**	0.3167	0.7268
		PHOSER	0.6375	0.4833	**0.7916**	30.83	0.2890	0.7187
		MFPSP	**0.7542**	**0.7333**	0.7750	4.17	**0.5088**	**0.8137**
	*M*. *oryzae*	NetPhos	0.5444	0.3888	0.7000	31.12	0.0935	0.5746
		NetPhosYeast	0.6500	0.7333	0.5666	16.67	0.3042	0.7044
		PHOSER	0.7333	0.5555	**0.9111**	35.56	0.4992	0.8019
		MFPSP	**0.8167**	**0.8333**	0.8000	**3.33**	**0.6337**	**0.8528**
	*N*. *crassa*	NetPhos	0.5954	0.3919	**0.7989**	40.7	0.2090	0.6767
		NetPhosYeast	0.6884	0.6783	0.6984	**2.01**	0.3769	0.7510
		PHOSER	0.6256	0.4572	0.7939	33.67	0.2668	0.7199
		MFPSP	**0.8015**	**0.8141**	0.7889	2.52	**0.6032**	**0.9055**
	*S*. *cerevisiae*	NetPhos	0.5694	0.6686	0.4701	19.85	0.1416	0.5869
		NetPhosYeast	0.6382	0.4609	**0.8156**	35.47	0.2958	0.7039
		PHOSER	0.6909	0.6980	0.6838	1.42	0.3820	0.7423
		ScerePhosSite	**0.7573**	**0.7507**	**0.7639**	1.32	**0.5147**	**0.8269**
		MFPSP	0.7426	0.7477	0.7376	1.01	0.4853	0.8105
	*S*. *pombe*	NetPhos	0.5755	0.3953	0.7558	36.05	0.1620	0.6074
		NetPhosYeast	0.6744	**0.8023**	0.5465	25.58	0.3608	0.7337
		PHOSER	0.7034	0.5930	**0.8139**	22.09	0.4172	0.7743
		MFPSP	**0.7849**	0.7674	0.8023	**3.49**	**0.5701**	**0.8327**
Y	*S*. *cerevisiae*	NetPhos	0.5274	0.5274	0.5274	**0.00**	0.0549	0.5477
		PHOSER	0.5769	0.5714	0.5824	1.10	0.1538	0.6106
		ScerePhosSite	0.6630	0.6700	0.6556	1.44	0.3260	0.7451
		MFPSP	**0.7106**	**0.7070**	**0.7143**	0.73	**0.4213**	**0.7525**

## Conclusion

In this study, a novel tool called MFPSP was developed for phosphorylation site prediction in multi-fungal species. The sequence information was extracted by physicochemical features and distributed information, furthermore, an offspring competition-based genetic algorithm was applied for selecting the optimal feature subset. Independent testing demonstrated that our proposed model achieves a more advanced and balanced performance as compared to several state-of-the-art available toolkits. Feature intersection, contribution and patterns were interpreted. The minus of MFPSP is that the sequence information is mainly based on sequence physicochemical and embedding features, exploring more advanced features will further elevate the performance and broaden the applications, for example, encoding sequences into images using chaos game representation [45] and Hilbert curve[46], extracting structure information by graph neural network [47–49], as well as 3D-structure information and large language models [50–52]. We anticipant MFPSP will supplements hands-on experiments by pre-screening potential phosphorylation sites and enhances our functional understanding of phosphorylation modification in fungi.

## Supporting information

S1 Methods *Physicochemical features methods*. (DOCX)

S1 Table Descriptor parameter search range and the best values. (DOCX)

S2 Table Hyperparameters search range for the four traditional classifiers. (DOCX)

S3 Table Thirteen types of physicochemical properties that used for computing the features of CTDC. (DOCX)

S4 Table Optimal parameters k and w for different species. (DOCX)

S5 Table Features and algorithms used in three compared methods. (DOCX)

S1 Fig Feature intersection of S phosphorylation among five fungi species. (DOCX)

S2 Fig SHAP dependence plots. (DOCX)
