# Supplementary material for: MFPSP: Identification of fungal species-specific phosphorylation site using offspring competition-based genetic algorithm
Source: PLoS Comput Biol. 2024 Nov 18;20(11):e1012607. doi: 10.1371/journal.pcbi.1012607 (PMC11611262; doi:10.1371/journal.pcbi.1012607)
Supplement: S1 Methods — (DOCX) [file pcbi.1012607.s001.docx]

**Supplementary methods**

*physicochemical features methods.*

Six feature descriptors for sequence feature representation. Amino acid composition (AAC), C/T/D-composition (CTDC), enhanced amino acid composition (EAAC), pseudo-amino acid composition (PAAC), quasi-sequence-order (QSOrder) and BINARY.

AAC calculates the frequencies of all 20 amino acids in a protein sequence (Bhasin and Raghava, 2004; Liu, 2019).

PAAC introduces a discrete model derived from the amino acid sequence to represent its sequence-order or pattern information. The PAAC descriptors(Chou, 2005) can be defined as follows:

Denoting the original hydrophobicity values of the 20 amino acids as $H_{1}^{O}$(i) (i = 1, 2, 3, …, 20). Similarly, the original hydrophobicity values and the original hydrophilicity values were denoted as $H_{2}^{O}$(i) and $M^{O}\left( i \right)$, respectively. They are transformed to the following quantities:

$\left\{ \begin{aligned} H_{1}\left( i \right)=\frac{H_{1}^{O}\left( i \right)-\frac{1}{20}\sum_{i=1}^{20} H_{1}^{O}\left( i \right)}{\sqrt{\frac{\sum_{i=1}^{20} \left[ H_{1}^{O}\left( i \right)-\frac{1}{20}\sum_{i=1}^{20} H_{1}^{O}\left( i \right) \right]^{2}}{20}}}, i=1,2,3,\ldots,20 \\ H_{2}\left( i \right)=\frac{H_{2}^{O}(i)-\frac{1}{20}\sum_{i=1}^{20} H_{2}^{O}(i)}{\sqrt{\frac{\sum_{i=1}^{20} \left[ H_{2}^{O}(i)-\frac{1}{20}\sum_{i=1}^{20} H_{2}^{O}(i) \right]^{2}}{20}}}, i=1,2,3,\ldots,20 \\ M^{O}\left( i \right)=\frac{M^{O}\left( i \right)-\frac{1}{20}\sum_{i=1}^{20} M^{O}\left( i \right)}{\sqrt{\frac{\sum_{i=1}^{20} \left[ M^{O}\left( i \right)-\frac{1}{20}\sum_{i=1}^{20} M^{O}\left( i \right) \right]^{2}}{20}}}, i=1,2,3,\ldots,20 \end{aligned} \right.$ (13)

$\Theta\left( R_{i},R_{j} \right)=\frac{1}{3}\{\left[ H_{1}\left( R_{i} \right)-H_{1}\left( R_{j} \right) \right]^{2}+\left[ H_{2}\left( R_{i} \right)-H_{2}\left( R_{j} \right) \right]^{2}+\left[ M\left( R_{i} \right)-M\left( R_{j} \right) \right]^{2}\}$ (14)

$\theta_{\lambda}=\frac{1}{N-\lambda}\sum_{i=1}^{N-\lambda} \Theta(R_{i},R_{i+\lambda})$ (15)

$X_{c}=\frac{f_{c}}{\sum_{r=1}^{20} f_{r}+w\sum_{j=1}^{\lambda} \theta_{j}}, (1\leq c\leq20)$ (16)

$X_{c}=\frac{w\theta_{c-20}}{\sum_{r=1}^{20} f_{r}+w\sum_{j=1}^{\lambda} \theta_{j}}, (21\leq c\leq20+\lambda)$ (17)

where $H_{k}\left( R_{i} \right)$ denotes the kth property of the amino acid $R_{i}$ in the amino acid property set, λ (λ < N) is an integer parameter that is chosen; $f_{c}$ is the normalized occurrence of the amino acids, weighting factor w = 0.05, and N is the sequence length. The descriptor dimension will be 20+λ.

The composition (C) feature (Govindan and Nair, 2011) characterizes the amino acid distribution patterns or physicochemical property in a protein. Twenty amino acids are categorized into three groups according to their physicochemical property (supplementary Table S3). Taking the charge attribute for example, twenty amino acids are categorized into positive group (KR), neutral group (ANCQGHILMFPSTWYV) and negative group (DE). The three features of the composition descriptor represent the percentage of each group of residues in the protein sequence and is calculated as follows:

$\mathrm{CTDC}\left( r \right)=\frac{N(r)}{N}, r\in\{postive, neutral, negative\}$ (7)

where $N(r)$ is the number of amino acids of type r in a given sequence and N is the protein length (Supplementary Table S3).

The first 20 features (Equation 8) of the QSOrder represents the amino acid frequency, and the remaining features characterize the sequence order based on the Schneider-Wrede physicochemical distance matrix (Schneider and Wrede, 1994) and the Grantham chemical distance matrix (Grantham, 1974) (Equation 9). It is defined as:

$X_{r}=\frac{f_{r}}{\underset{r=1}{\overset{20}{\sum}}f_{r}+w\underset{d=1}{\overset{nlag}{\sum}}\tau_{d}}, r=1, 2, 3, \ldots, 20$ (8)

$X_{d}=\frac{w\tau_{d-20}}{\underset{r=1}{\overset{20}{\sum}}f_{r}+w\underset{d=1}{\overset{nlag}{\sum}}\tau_{d}}, d=21,2 2, 23, \ldots,nlag$ (9)

$\tau_{d}=\sum_{i=1}^{N-d} {(d_{i,i+d})}^{2}, d=1, 2,3, \ldots, nlag$ (10)

where $f_{r}$ is the normalized occurrence of amino acid type r and weighting factor w = 0.1; $d_{i,i+d}$ is the distance between the two amino acids at position i and i + d in protein sequence; *nlag* is the maximum value of the lag, N is the protein length. Accordingly, the descriptor dimension will be 40+2×nlag.

The Enhanced Amino Acid Composition (EAAC) calculates the AAC based on the sequence window of fixed length that continuously slides from the N- to C-terminus of each peptide and can be usually applied to encode the peptides with an equal length(Chen, et al., 2018).

In the Binary encoding each amino acid is represented by a 20-dimensional binary vector, e.g. A is encoded by (10000000000000000000), C is encoded by (01000000000000000000), …, Y is encoded by (00000000000000000001), respectively(Chen, et al., 2018).

*Machine learning methods*

The light gradient boosting machine (LGB) were used for parameter optimization of Qsorder, EAAC, and PAAC, the ACC were used to select the best values as listed in supplementary table S1. The SVM algorithm was used at the combined physicochemical-feature selection based on ACC. For embedding feature selection, the optimal K and W were evaluated by SVM based on ACC. At last, the combined physicochemical and embedding features were subjected to feature selection by genetic algorithm in which the LGB were used for fitness calculation based on ACC.

**References**

1. Bhasin, M. and Raghava, G.P.S. Classification of nuclear receptors based on amino acid composition and dipeptide composition. *Journal of Biological Chemistry* 2004;279(22):23262-23266.

2. Chen, Z.*, et al.* iFeature: a python package and web server for features extraction and selection from protein and peptide sequences. *Bioinformatics* 2018;34(14):2499-2502.

3. Chou, K.C. Using amphiphilic pseudo amino acid composition to predict enzyme subfamily classes. *Bioinformatics* 2005;21(1):10-19.

4. Govindan, G. and Nair, A.S. Composition, transition and distribution (CTD)-A dynamic feature for predictions based on hierarchical structure of cellular sorting. In, *Annual IEEE India Conference - Engineering Sustainable Solutions*. BITS Pilani, Hyderabad Campus, Hyderabad, INDIA; 2011.

5. Grantham, R. Amino acid difference formula to help explain protein evolution. *Science (New York, N.Y.)* 1974;185(4154):862-864.

6. Liu, B. BioSeq-Analysis: a platform for DNA, RNA and protein sequence analysis based on machine learning approaches. *Briefings in Bioinformatics* 2019;20(4):1280-1294.

7. Schneider, G. and Wrede, P. The rational design of amino-acid-sequences by artificial neural networks and simulated molecular evolution - de-novo design of an idealized leader peptidase cleavage site. *Biophysical Journal* 1994;66(2):335-344.
