# Supplementary material for: MFPSP: Identification of fungal species-specific phosphorylation site using offspring competition-based genetic algorithm
Source: PLoS Comput Biol. 2024 Nov 18;20(11):e1012607. doi: 10.1371/journal.pcbi.1012607 (PMC11611262; doi:10.1371/journal.pcbi.1012607)
Supplement: S1 Table — (DOCX) [file pcbi.1012607.s002.docx]

**S1 Table** Descriptor parameter search range and the best values

| **Feature encodings** | **Parameters** | **Search range** | **Optimal value** |
| --- | --- | --- | --- |
| QSorder | nlag | [1,2,3,…,10] | 1 |
| PAAC | λ | [1,2,3,…,10] | 2 |
| EAAC | w | [1,2,3,…,10] | 5 |
