# Supplementary material for: MFPSP: Identification of fungal species-specific phosphorylation site using offspring competition-based genetic algorithm
Source: PLoS Comput Biol. 2024 Nov 18;20(11):e1012607. doi: 10.1371/journal.pcbi.1012607 (PMC11611262; doi:10.1371/journal.pcbi.1012607)
Supplement: S2 Table — (DOCX) [file pcbi.1012607.s003.docx]

**S2 Table** Hyperparameters search range for the three traditional classifiers.

| **Method** | **Parameter grid search ranges** |
| --- | --- |
| Random Forest (RF) | n_estimators: [10, 20, …, 200]  max_features: [1, 2, 3, …,10]  min_samples_split: [1, 2, 3, …,10] |
| Support Vector Machine (SVM) | C: [0.01, 0.05, 0.1, 0.5, 1, 5, 10, …, 100]  gamma: [0.0001, 0.0002, 0.0004, …, 0.1, 0.2, 0.4, …, 8]  Kernel: [rbf] |
| Light gradient boosting machine (LGB) | n_estimators: [10, 20, …, 200]  learning_rate: [0.01, 0.05, 0.1]  max_depth: [2, 4, 6, 8]  num_leaves: [10, 20, 30, …, 100] |
