# Supplementary material for: MFPSP: Identification of fungal species-specific phosphorylation site using offspring competition-based genetic algorithm
Source: PLoS Comput Biol. 2024 Nov 18;20(11):e1012607. doi: 10.1371/journal.pcbi.1012607 (PMC11611262; doi:10.1371/journal.pcbi.1012607)
Supplement: S3 Table — (DOCX) [file pcbi.1012607.s004.docx]

**S3 Table** Thirteen types of physicochemical properties that used for computing the features of CTDC.

| **physicochemical properties** | **categorized groups** | | |
| --- | --- | --- | --- |
| Hydrophobicity_PRAM900101 | Polar: RKEDQN | Neutral: GASTPHY | Hydrophobicity: CLVIMFW |
| Hydrophobicity_ARGP820101 | Polar: QSTNGDE | Neutral: RAHCKMV | Hydrophobicity: LYPFIW |
| Hydrophobicity_ZIMJ680101 | Polar: QNGSWTDERA | Neutral: HMCKV | Hydrophobicity: LPFYI |
| Hydrophobicity_PONP930101 | Polar:KPDESNQT | Neutral: GRHA | Hydrophobicity:YMFWLCVI |
| Hydrophobicity_CASG920101 | Polar:KDEQPSRNTG | Neutral: AHYMLV | Hydrophobicity: FIWC |
| Hydrophobicity_ENGD860101 | Polar:RDKENQHYP | Neutral :SGTAW | Hydrophobicity: CVLIMF |
| Hydrophobicity_FASG890101 | Polar: KERSQD | Neutral: NTPG | Hydrophobicity:AYHWVMFLIC |
| Normalized van der Waals volume | Volume range: 0-2.78  GASTPD | Volume range: 2.95-94.0  NVEQIL | Volume range: 4.03-8.08  MHKFRYW |
| Polarity | Polarity value:4.9-6.2  LIFWCMVY | Polarity value: 8.0-9.2  PATGS | Polarity value: 10.4-13.0  HQRKNED |
| Polarizability | Polarizability value: 0-1.08  GASDT | Polarizability value:0.128-120.186  GPNVEQIL | Polarizability value: 0.219-0.409  KMHFRYW |
| Charge | Positive: KR | Neutral:ANCQGHILMFPSTWYV | Negative: DE |
| Secondary structure | Helix:EALMQKRH | Strand: VIYCWFT | Coil: GNPSD |
| Solvent accessibility | Buried:ALFCGIVW | Exposed: PKQEND | Intermediate: MPSTHY |
