# Supplementary material for: MFPSP: Identification of fungal species-specific phosphorylation site using offspring competition-based genetic algorithm
Source: PLoS Comput Biol. 2024 Nov 18;20(11):e1012607. doi: 10.1371/journal.pcbi.1012607 (PMC11611262; doi:10.1371/journal.pcbi.1012607)
Supplement: S4 Table — (DOCX) [file pcbi.1012607.s005.docx]

**S4 Table** Optimal parameters k and w for different species

| Species | Site type | Search range | Optimal k an w |
| --- | --- | --- | --- |
| *Aspergillus sp.^*^* | S | k: k-mer length (2-10)  w: window size (1-10) | k=7, w=1 |
|  | T |  | k=7, w=1 |
|  | Y |  | — |
| *C. neoformans* | S |  | k=7, w=2 |
|  | T |  | — |
|  | Y |  | — |
| *F. graminearum* | S |  | k=7, w=1 |
|  | T |  | k=6, w=7 |
|  | Y |  | — |
| *M. oryzae* | S |  | k=7, w=1 |
|  | T |  | k=7, w=3 |
|  | Y |  | — |
| *N. crassa* | S |  | k=7, w=2 |
|  | T |  | k=7, w=1 |
|  | Y |  | — |
| *S. cerevisiae* | S |  | k=7, w=1 |
|  | T |  | k=7, w=1 |
|  | Y |  | k=9, w=1 |
| *S. pombe* | S |  | k=7, w=1 |
|  | T |  | k=7, w=4 |
|  | Y |  | — |
