# Supplementary material for: MFPSP: Identification of fungal species-specific phosphorylation site using offspring competition-based genetic algorithm
Source: PLoS Comput Biol. 2024 Nov 18;20(11):e1012607. doi: 10.1371/journal.pcbi.1012607 (PMC11611262; doi:10.1371/journal.pcbi.1012607)
Supplement: S5 Table — (DOCX) [file pcbi.1012607.s006.docx]

**S5 Table** Features and algorithms used in three compared methods

| **Methods** | **Features** | **Algorithms** | **Website** |
| --- | --- | --- | --- |
| NetPhos | structural information from PDB | Artificial Neural Network | https://services.healthtech.dtu.dk/services/NetPhos-3.1/ |
| NetPhosYeast | BLOSUM62 scoring matrix | Artificial Neural Network | http://www.cbs.dtu.dk/services/NetPhosYeast/ |
| PHOSER | AAindex | RF | https://saphire.usask.ca/saphire/phosfer/index.html |
| ScerePhoSite | amino acid composition (AAC), CTD composition (CTDC), enhanced amino acid composition (EAAC), quasi-sequence order (QSOrder) and pseudo amino acid composition (PAAC) | SVM | https://github.com/wangchao-malab/ScerePhoSite/ |
