# Supplementary material for: MFPSP: Identification of fungal species-specific phosphorylation site using offspring competition-based genetic algorithm
Source: PLoS Comput Biol. 2024 Nov 18;20(11):e1012607. doi: 10.1371/journal.pcbi.1012607 (PMC11611262; doi:10.1371/journal.pcbi.1012607)
Supplement: S1 Fig — (DOCX) [file pcbi.1012607.s007.docx]

**
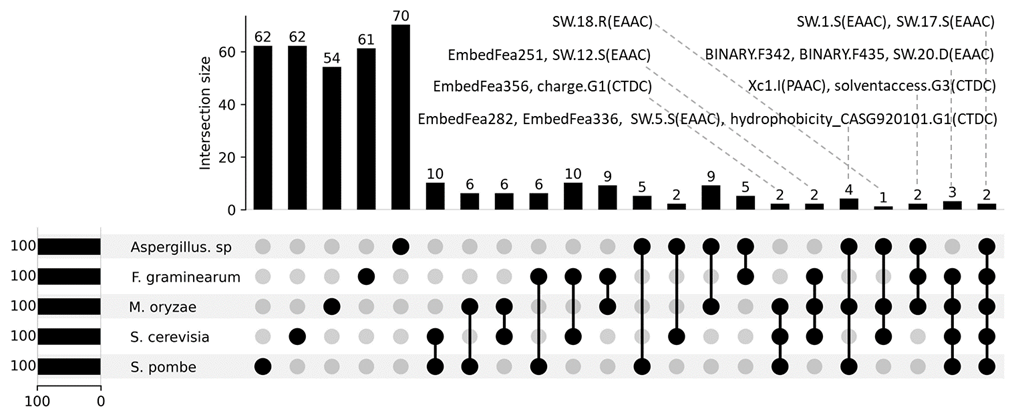
**

**S1 Fig.** Feature intersection of S phosphorylation among five fungi species. 16 features that presented at least in three species were labeled, two features, i.e, SW.1.S(EAAC) and SW.17.S(EAAC), were identified in all five species, three features, namely BINARY.F342, BINARY.F435 and SW.20.D(EAAC) were presented in four species.
