# Supplementary material for: MFPSP: Identification of fungal species-specific phosphorylation site using offspring competition-based genetic algorithm
Source: PLoS Comput Biol. 2024 Nov 18;20(11):e1012607. doi: 10.1371/journal.pcbi.1012607 (PMC11611262; doi:10.1371/journal.pcbi.1012607)
Supplement: S2 Fig — (DOCX) [file pcbi.1012607.s008.docx]

**
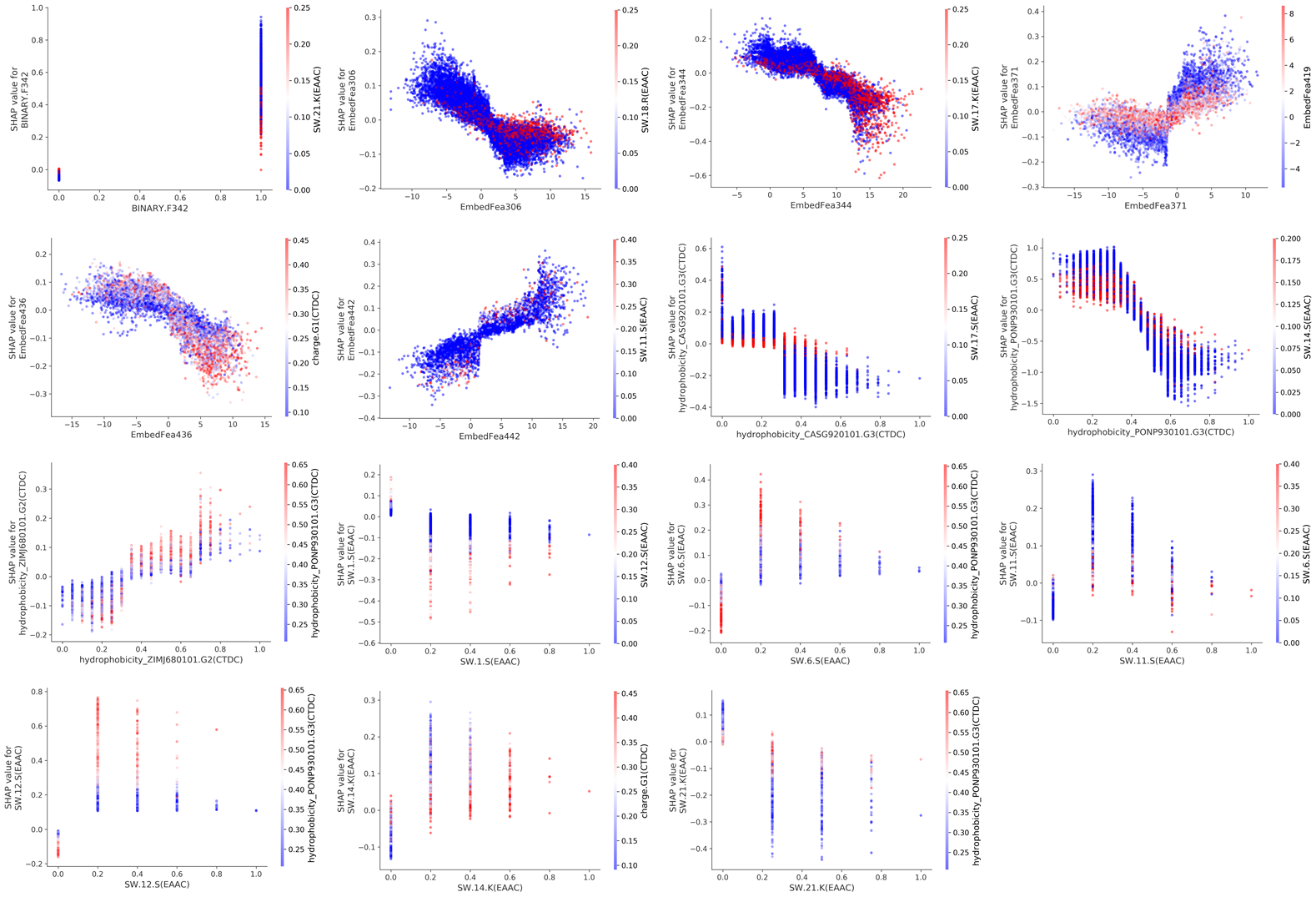
**

**S2 Fig**. SHAP dependence plots. These plots show the effect that a single feature has on the model and the interaction effects across features.
